# Pregnancy in Non-Communicating Rudimentary Horn of A Unicornuate Uterus

**DOI:** 10.22074/ijfs.2018.5022

**Published:** 2017-10-14

**Authors:** Mania Kaveh, Abolfazl Mehdizadeh Kashi, Kambiz Sadegi, Forough Forghani

**Affiliations:** 1 Endometriosis and Gynecological Disorder Research Center, Iran University of Medical Science, Tehran, Iran; 2 Department of Obstetrics and Gynecology, Zabol University of Medical Science, Zabol, Iran; 3 Pain Research Center, Iran University of Medical Science, Tehran, Iran; 4 Department of Anesthesiology, Zabol University of Medical Science, Zabol, Iran

**Keywords:** Pregnancy, Rudimentary, Uterus

## Abstract

Diagnosis and management of pre-rupture stage of the pregnant horn are difficult and usually missed on a routine ul- trasound scan. Also most cases are detected after rupture of pregnant horn. We presented a 28-year-oldG2 L1 woman with diagnosis of rudimentary horn pregnancy (RHP) at 14 weeks of gestation. We diagnosed her with a normal intrauterine pregnancy, whereas a pregnancy in a right-sided non-communicating rudimentary horn with massive he- moperitoneum was later discovered on laparotomy. RHP has a high risk of death for mother, so there must be a strong clinical suspicion for the diagnosis of RHP. Although there is a major advancement in field of diagnostic ultrasound and other imaging modalities, prenatal diagnosis has remained elusive and a laparotomy surgery is considered as a definitive diagnosis.

## Introduction

Rudimentary horn pregnancy (RHP) as a rare incidence, has been estimated at 1:76,000-1:160,000 pregnancies (1). It has also been reported that 75-83% of cases are the pregnancy in non-communicating rudimentary horn that is caused by transmigration of peritoneal sperm or fertilized ovum (2). Gynecological and obstetrical complications of pregnancy in unicornuate uterus with a rudimentary horn are as following: i. Spontaneous abortion, ii. Preterm labor, iii. Infertility, iv. Endometriosis, v. Hematometra, vi. Intrauterine growth restriction (IUGR), vii. Intra peritoneal bleeding, and viii.. Uterine rupture. Kidney abnormalities have also been reported in 31% of cases, while the patients were diagnosed after reaching their stable condition (3). Rupture of RHP is considered as a life threatening condition for mothers. We used timely laparotomy, excision of the horn and blood transfusion to save a 28-year-old G2 L1 woman who was initially diagnosed with a normal intrauterine pregnancy, but a 14-week pregnancy in a right-sided non-communicating rudimentary horn with massive hemoperitoneum was later discovered on laparotomy. 

## Case report

A 28-year-old G2L1 woman who was 14 weeks pregnant was admitted at Amiralmomenin Hospital, Zabol, Iran, in November 2015, with generalized abdominal pain, nausea and vomiting. The patient who had a previous cesarean section received early prenatal care two years ago and an ultrasound exam at 14 weeks of gestation (a day before she was admitted at the hospital). The patient suffering from hypovolemic shock, was extremely pale, and had a weak pulse of 120-130 beats per minute and a blood pressure of 80/60 mm Hg. Physical exam revealed impaired consciousness and agitation, generalized abdominal tenderness with sharp right lower quadrant (RLQ) pain, no vaginal bleeding, and a closed cervix. A portable ultrasound detected more than 2 liters of free fluid in the abdomen and pelvis that confirmed the presence of unicorn ate uterus and a 14-week pregnancy in right-sided rudimentary horn. After fluid resuscitation, the patient was transferred to the operating room for an emergency laparotomy. Her blood pressure was 90/60 mm Hg at the time of laparotomy. 

**Fig.1:**
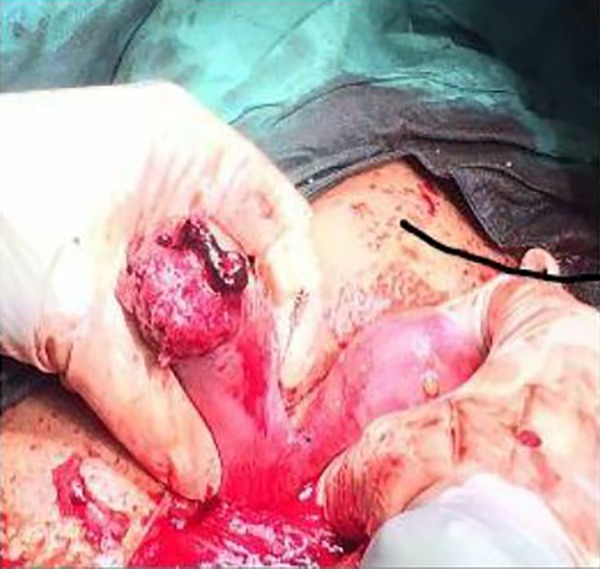
Rudimentary horn pregnancy still attached to the main horn.

**Fig.2:**
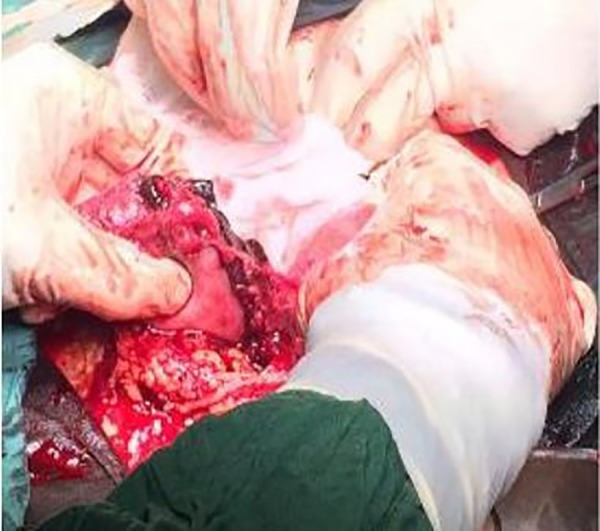
A 4-cm ruptured rudimentary horn with placenta partially protruding from it.

**Fig.3:**
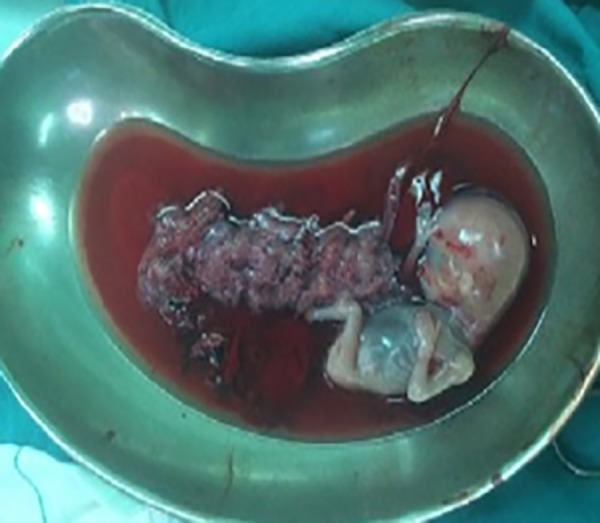
Fetus with placenta.

**Fig.4:**
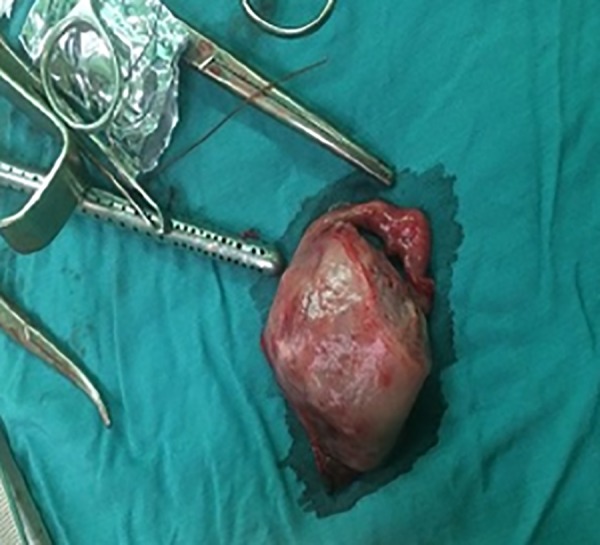
Excised rudimentary horn.

During laparotomy, we founded that right-sided noncommunicating rudimentary horn was already ruptured and the fetus with amniotic sac extruded into the peritoneal cavity with presence of about a 3-liter hemoperitoneum (Figs .1-3). The rudimentary horn was then excised (Fig .4) and the abdomen closed following hemostasis. Furthermore, patient received 3 unites of pack cell and recovered well after surgery. She was discharged with satisfactory condition on fifth post-operative day after the kidney anomalies ruled out. This study was approved by the Ethics Committee of Iran University of Medical Sciences, Tehran, Iran. Written informed consent was obtained from case. 

## Discussion

Pregnancy in a non-communicating rudimentary horn is the results of developmental defect of one Müllerian duct or incomplete connection with Müllerian ducts on the opposite site that has been estimated at 1:76,000-1:160,000 pregnancies (1,4). The first case of uterine rupture following RHP was reported by Kanagal and Hanumanalu (5). It has been reported that the timing of a ruptured rudimentary horn that is mainly associated with horn musculature and its ability to hypertrophy is estimated between 5 and 35 weeks. The early diagnosis of RHP is likely to prevent maternal morbidity and mortality. The best management strategies for early diagnosis of RHP are as: i. Ultrasound, ii. Hysterosalpingography, hysteroscopy, laparoscopy, as well as iii. Magnetic resonance imaging (MRI) (5). It is noted that the sensitivity of ultrasound is 26%, although its sensitivity decreases when the maternal age increases (6). 

However, RHP is likely to be missed by the most experienced radiologist. The most common ultrasound reports that leads to misdiagnose RHP are as follows: i. Tubal pregnancy, ii. Cornal pregnancy, iii. Intrauterine pregnancy, and iv. Abdominal pregnancy (7). It is difficult to confirm a rudimentary horn with thin myometrium diagnosis because of obscuring adjacent anatomical structures. The following diagnostic criteria for RHP were indicated by Tsafrir et al. (8) using ultrasonography: i. Pseudo pattern of asymmetrical bicornuate uterus, ii. Non-continuity between tissue surrounding the gestational sac and the uterine cervical canal, and iii. The presence of myometrial tissue surrounding the gestational sac. Furthermore, a hyper vascularization pattern like placenta accrete is considered as an indication for the diagnosis of RHP that is detected by both Color Doppler ultrasound and Doppler ultrasound. Samuels and Awonuga (9) have reported a uterine rupture after labor induction with misoprostol. 

The application of different methods of labor induction for termination of RHP was unsuccessful and led to a uterine rupture. The surgical approach is considered as the first management strategy. There are several reports of early diagnosis and laparoscopic excision of rudimentary horn (10,12). Edelman has reported a successful strategy treatment of RHP including the use of methotrexate (MTX) and laparoscopic excision of rudimentary horn in the first weeks of pregnancy (13). Emergency surgery after diagnosis even in cases of un-ruptured rudimentary horn has been recommended (3). Also, prophylactic removal of the rudimentary horn has been suggested (14). There is a report of RHP reaching a full-term delivery that led to live birth using cesarean section (15). The reproductive outcome of a unicornuate uterus is discussed in some articles. However, afew of them have discussed the reproductive outcome after resection of rudimentary horn. Those patients who have ever undergone resection of a rudimentary horn should be considered as a high-risk group in the fallowing pregnancy (16,17). This case report had no ethical consideration for patient. 

## Conclusion

RHP has high risk of death for mother, so there must be a strong clinical suspicion for the diagnosis of RHP. Although there is a major advancement in field of diagnostic ultrasound and other imaging modalities, prenatal diagnosis has remained elusive and a laparotomy surgery is considered as a definitive diagnosis. Early diagnosis, timely resuscitation, laparotomy, and blood transfusion are the necessary management steps to save a patient. 
